# Novel Properties of Old Propranolol—Assessment of Antiglycation Activity through *In Vitro* and *In Silico* Approaches

**DOI:** 10.1021/acsomega.4c03025

**Published:** 2024-06-11

**Authors:** Kamil Klaudiusz Lauko, Miłosz Nesterowicz, Daria Trocka, Karolina Dańkowska, Małgorzata Żendzian-Piotrowska, Anna Zalewska, Mateusz Maciejczyk

**Affiliations:** † ‘Biochemistry of Civilisation Diseases’ Students’ Scientific Club at the Department of Hygiene, Epidemiology and Ergonomics, Medical University of Bialystok, 2c Mickiewicza Street, Bialystok 15-233, Poland; ‡ Department of Hygiene, Epidemiology and Ergonomics, Medical University of Bialystok, 2c Mickiewicza Street, Bialystok 15-233, Poland; § Independent Laboratory of Experimental Dentistry, Medical University of Bialystok, 24a M. Sklodowskiej-Curie Street , Bialystok 15-274, Poland

## Abstract

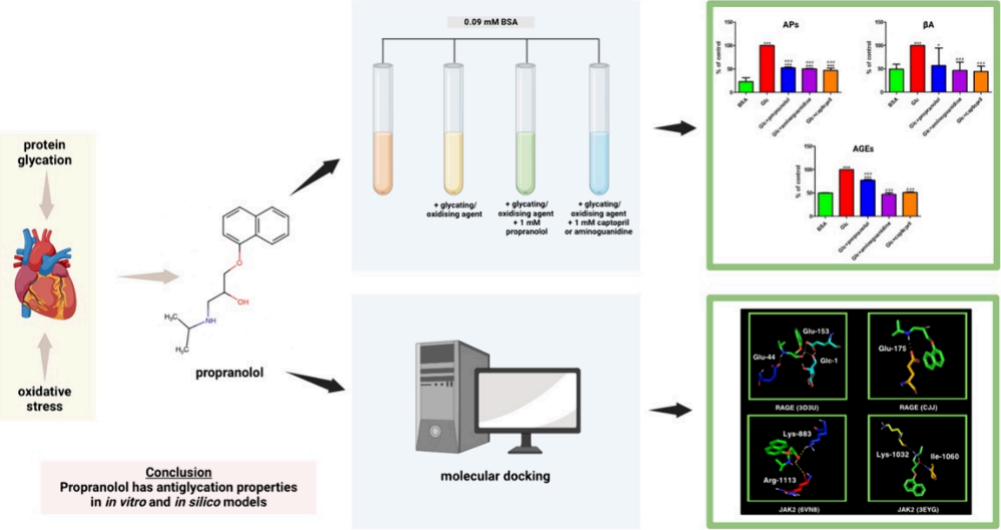

Hypertension has earned the “silent killer” nickname since it may lead to a number of comorbidities, including diabetes and cardiovascular diseases. Oxidative stress and protein glycation play vital roles in the pathogenesis of hypertension. Several studies have shown that they profoundly account for vascular dysfunction, endothelial damage, and disruption of blood pressure regulatory mechanisms. Of particular note are advanced glycation end products (AGEs). AGEs alter vascular tissues’ functional and mechanical properties by binding to receptors for advanced glycation end products (RAGE), stimulating inflammation and free radical-mediated pathways. Propranolol, a nonselective beta-adrenergic receptor antagonist, is one of the most commonly used drugs to treat hypertension and cardiovascular diseases. Our study is the first to analyze propranolol’s effects on protein glycoxidation through *in vitro* and *in silico* approaches. Bovine serum albumin (BSA) was utilized to evaluate glycoxidation inhibition by propranolol. Propranolol (1 mM) and BSA (0.09 mM) were incubated with different glycating (0.5 M glucose, fructose, and galactose for 6 days and 2.5 mM glyoxal and methylglyoxal for 12 h) or oxidizing agents (chloramine T for 1 h). Biomarkers of protein glycation (Amadori products (APs), β-amyloid (βA), and advanced glycation end products (AGEs)), protein glycoxidation (dityrosine (DT), kynurenine (KYN), and *N*-formylkynurenine (NFK)), protein oxidation (protein carbonyls (PCs), and advanced oxidation protein products (AOPPs)) were measured by means of colorimetric and fluorimetric methods. The scavenging of reactive oxygen species (hydrogen peroxide, hydroxyl radical, and nitric oxide) and the antioxidant capacity (2,2-diphenyl-1-picrylhydrazyl radical and ferrous ion chelating (FIC) assays)) of propranolol were also evaluated. Additionally, *in silico* docking was performed to showcase propranolol’s interaction with BSA, glycosides, and AGE/RAGE pathway proteins. The products of protein glycation (↓APs, ↓βA, ↓AGEs), glycoxidation (↓DT, ↓KYN, ↓NFK), and oxidation (↓PCs, ↓AOPPs) prominently decreased in the BSA samples with both glycating/oxidizing factors and propranolol. The antiglycoxidant properties of propranolol were similar to those of aminoguanidine, a known protein oxidation inhibitor, and captopril, which is an established antioxidant. Propranolol showed a potent antioxidant activity in the FIC and H_2_O_2_ scavenging assays, comparable to aminoguanidine and captopril. *In silico* analysis indicated propranolol’s antiglycative properties during its interaction with BSA, glycosidases, and AGE/RAGE pathway proteins. Our results confirm that propranolol may decrease protein oxidation and glycoxidation *in vitro*. Additional studies on human and animal models are vital for *in vivo* verification of propranolol’s antiglycation activity, as this discovery might hold the key to the prevention of diabetic complications among cardiology-burdened patients.

## 1 Introduction

Hypertension is a significant predictor of increased morbidity and mortality in patients throughout the world.^1^ Primary hypertension is directly connected to total peripheral resistance and cardiac output originated by modifiable risk factors, e.g., physical inactivity, smoking, excessive alcohol intake, a diet high in sodium, and psychological stress.^2−4^ Consequently, hypertension is a civilizational disease. It is diagnosed more often in males than in females for individuals below 65 years of age.^5^ Hypertension is a significant risk factor for cardiovascular diseases, strokes, kidney diseases, and hypertensive retinopathy. The prevalence of hypertension has risen 2-fold to reach 1.28 billion individuals since 1990.^6^ Treatment of hypertension is considered the most common reason for visits to general practitioners and for the prescription of chronic medications.^7^ The most common hypertensive drugs are beta-blockers, thiazide and thiazide-like diuretics, angiotensin-converting enzyme (ACE) inhibitors, angiotensin receptor blockers (ARBs), calcium channel blockers (CCBs), and alpha-blockers.^8^

**Figure 1:**
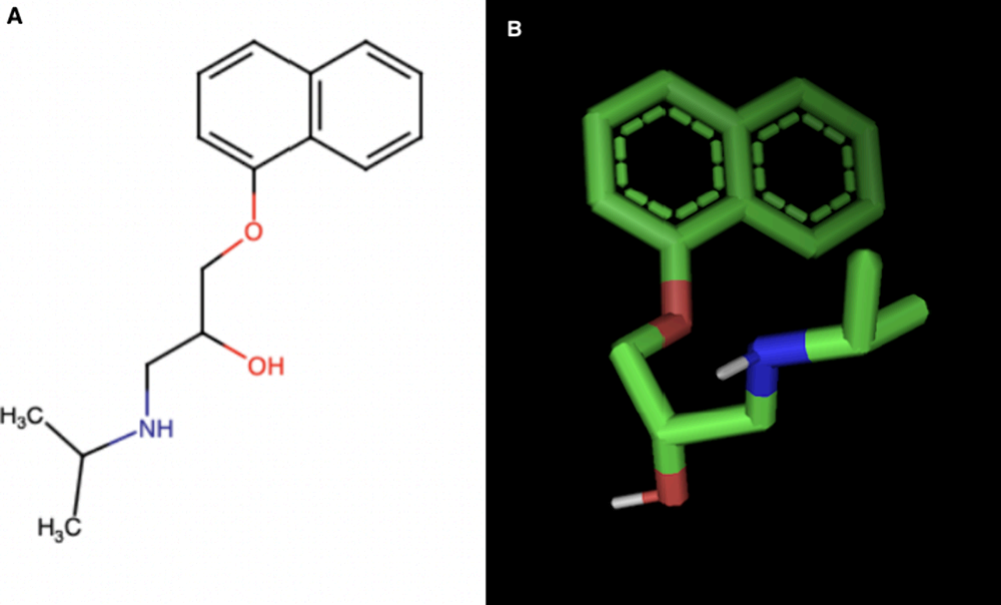
Structural formula (A) and the spatial structure (B) of propranolol (1-naphthalen-1-yloxy-3-(propan-2-ylamino)propan-2-ol;hydrochloride).

Propranolol (C_16_H_21_NO_2_; 1-naphthalen-1-yloxy-3-(propan-2-ylamino)propan-2-ol; Figure 1) is a nonselective beta-adrenergic antagonist used for treating hypertension, angina pectoris, myocardial infarction, migraine, pheochromocytoma, cardiac arrhythmias, and hypertrophic cardiomyopathy.^9^ It competitively blocks beta_1_- and beta_2_-adrenergic stimulation, which decreases the blood pressure, heart rate, myocardial contractility, and myocardial oxygen demand. Propranolol also reduces portal pressure by producing splanchnic vasoconstriction (beta_2_ effect). After oral administration, the drug is absorbed rapidly and completely.^10^ The onset of actions is 1 to 2 h after administration, and the peak effect is usually seen within a few days to several weeks. The main action of propranolol is mainly due to its parent compound. Propranolol is metabolized by cytochrome P450 in the liver; however, propranolol’s metabolites exhibit significantly lower activity than the parent compound.^11^

Protein glycation, particularly the formation of advanced glycation end products (AGEs), plays a vital role in the evolution and advancement of cardiovascular diseases. Protein glycation profoundly impacts endothelial cell function, as it compromises the inner lining of blood vessels. It leads to reduced vasodilation, increased vascular permeability, and cellular inflammation, leading to atherosclerosis.^12^ AGEs also play a crucial role in myocardial damage by being accumulated in the heart tissues and thus affecting the structure and function of cardiac proteins.^13^ AGE accumulation may impact myocardial fibrosis and contractility impairment and increase the likelihood of arrhythmia.^14^ On the molecular level, AGEs activate specific receptors, mainly for advanced glycation end products (RAGE), thereby triggering pro-inflammatory and oxidative stress pathways.^15^

Drugs with antiglycoxidative effects are particularly favored in cardiology. Nevertheless, little is known about the antioxidant and antiglycation properties of propranolol. The studied literature/current data are inconclusive, and thus, we are the first to investigate propranolol for its antiglycoxidative activity using various *in vitro* and *in silico* models. We have also conducted a systematic literature review on the antiglycation properties of propranolol.

## 2 Materials and Methods

### 2.1 Systematic Review

The literature review was performed between 1995 and 2023 on the Medline (PubMed) database. The available bibliography was studied by using the following keywords: [propranolol and antiglycoxidative properties], [propranolol and antiglycation properties], [propranolol and antioxidative properties], [propranolol and oxidative stress], [propranolol and carbonyl stress], [propranolol and protein glycation], [propranolol and nitrosative stress], and [propranolol and ROS scavenging]. Inclusion and exclusion criteria are demonstrated in Table 1.

**Table 1:** Inclusion and Exclusion Criteria of the Examined Publications

**inclusion criteria**	**exclusion criteria**
publications written in English	publications written in other languages
manuscripts relevant to human/animal *in vivo* and *in vitro* experiments	review papers, surveys, and case descriptions
articles on antiglycooxidative activity of propranolol	articles not describing the antiglycooxidative activity of propranolol

**Figure 2:**
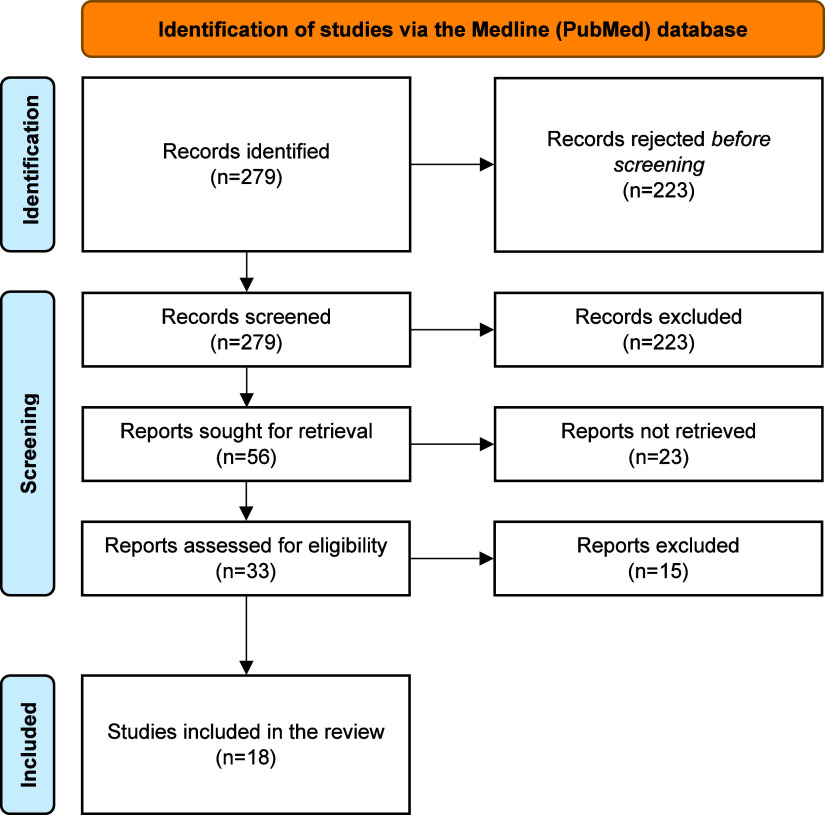
Flowchart in accordance with PRISMA guidelines: the systematic review methodology.

At first, initial data was investigated by analyzing titles and abstracts of publications independently by two researchers (K.K.L, M.N.). Then, two other researchers inspected all of the previously extracted manuscripts (M.N., D.T.). Only the papers compliant with the inclusion and exclusion criteria were employed for the final analysis. The Cohen’s kappa coefficient (κ) was calculated to measure the level of the researcher’s reliability. The result was κ = 0.94. Every article was evaluated methodologically, with the following elements undergoing the analyses: authors, publication year, study design, experiment population size, inclusion and exclusion criteria, length of research, and end points (Figure 2).

### 2.2 Reagents and Equipment

All of the analytical grade reactants were obtained from Sigma-Aldrich (Numbrecht, Germany/St. Louis, Missouri, USA). 0.2 mm membrane filters were used for sterilizing all chemical solutions promptly before utilization. An M200 PRO multimode microplate reader (Tecan Group, Ltd., Männedorf, Switzerland) assayed the absorbance and fluorescence.

## 3 Scavenging of Reactive Oxygen Species (ROS)

### 3.1 Hydrogen Peroxide (H_2_O_2_) Scavenging Capacity

The method recommended by Kwon et al. was implemented to assess the H_2_O_2_ scavenging activity.^16^ First of all, 87.3 mg of butylated hydroxytoluene (BHT), 10 μL of sulfuric acid (H_2_SO_4_), 7.6 mg of xylenol orange, and 10 mg of ferrous ammonium sulfate were amalgamated in 100 mL of 90% methanol–water solution to acquire a solution of ferrous ion oxidation-xylenol orange (FOX). Afterward, H_2_O_2_ (50 mM) as well as the samples (final concentration: 1 mM) were mixed (1:1 and v/v) and next incubated for 30 min in room temperature conditions. Later, high-performance liquid chromatography (HPLC)-grade methanol (10 μL) was added to the sample solution (90 μL) in H_2_O_2_. Next, FOX reagent (0.9 mL) was mixed with the produced mixture, vortexed, and incubated at room temperature for 30 min. The reaction product (ferric ion-xylenol orange) was assayed spectrophotometrically at a wavelength of 560 nm. The scavenging of H_2_O_2_ (%) was quantified using the formula [1 – *f* (*A*_1_ – *A*_2_)/A_0_] × 100%, where *A*_0_ represents the control absorbance (without added drugs), *A*_1_ represents the absorbance following the addition of the drugs, and *A*_2_ represents the absorbance without the addition of FOX reagent.^16^

### 3.2 Hydroxyl Radical (HO•) Scavenging Capacity

The assay modified by Su et al. was implemented to measure the scavenging activity of HO•.^17^ 0.25 mL of ferrous sulfate (FeSO_4_), 0.4 mL of hydrogen peroxide (H_2_O_2_) (6 mM), 0.25 mL of distilled water (H_2_O), 0.5 mL of the samples (final concentration: 1 mM), and 0.2 mL of sodium salicylate (C_7_H_5_NaO_3_) (20 mM) were all mixed to be subsequently incubated for 1 h at 37 °C.^17^ At the 562 nm wavelength, the reaction mixture’s absorbance was measured. Next, the following formula was employed to count the scavenged HO• (%) by means of the formula [1 – *f*(*A*_1_ – *A*_2_)/*A*_0_] × 100%, where *A*_0_ represents the control absorbance (without added drugs), *A*_1_ represents the absorbance following the addition of the drugs, and *A*_2_ represents the absorbance without the addition of sodium salicylate.^17^

### 3.3 2,2-Diphenyl-1-picrylhydrazyl (DPPH•) Scavenging Capacity

The method recommended by Kwon et al. was followed to determine the free radical scavenging activity by means of decolorization of the DPPH radical.^16^ Summarily, the diluted sample (30 μL) was mixed with the DPPH**•** solution (0.13 mg/mL) (180 μL), after which the solution was adjusted to a final volume of 210 μL by adding methanol. The DPPH solution assisted as a control. Then, the reaction mixture was incubated for 20 min at room temperature and the absorbance of the reaction mixture was measured at a 518 nm wavelength using a spectrophotometer. The inhibition rate (%) was calculated using the formula [(*A*_blank_ – *A*_sample_)/*A*_blank_] × 100%, where *A*_blank_ represents the absorbance of the blank DPPH solution and *A*_sample_ represents the DPPH solution after the addition of the sample.^16^

### 3.4 Nitric Oxide (NO•) Scavenging

First, 100 μL of phosphate-buffered saline (PBS) containing 5 mM sodium nitroprusside (SNP) was added to each 50 μL sample. The mixture was next incubated at 25 °C for 150 min. Next, 155 μL of Griess reagent containing 1% of sulfanilamide, 2% of phosphoric acid (H_3_PO_4_), and 0.1% of *N*-(1-naphthyl) ethylenediamine were added to the reaction mixture. A chromophore was released through nitrite diazotization of sulfanilamide with its conjugation with *N*-(1-naphthyl) ethylenediamine. The absorbance of the reaction product was determined spectrophotometrically at a 546 nm wavelength. The scavenged NO• (%) was calculated using the formula [1 – (*A*_1_/*A*_2_)] × 100%, where *A*_1_ represents the sample absorbance (with the addition of drugs) and *A*_2_ represents the absorbance without Griess reagent.^18^

### 3.5 Ferrous Ion Chelation (FIC)

FIC activity was determined by measuring the decrease in the formation of the Fe^2+^–ferrozine complex. FeCl_2_ (18 μL, 0.06 mM) and CH_3_OH (16 μL) were mixed together with the samples (90 μL, terminal strength of 1 mM) or the BHT control. Immediately afterward, a ferrozine solution (18 μL, 5 mM) was added in order for the mixture to be incubated for an extra 5 min at room temperature. A spectrophotometer was used for measuring the absorbance at a wavelength of 562 nm. The percentage decrease in absorbance as compared to the control was then calculated to determine the ferrous ion chelating (FIC) activity.^16^

## 4 Bovine Serum Albumin (BSA) Model

Glycation/oxidation of BSA was performed based on previously applied methods.^19−25^ BSA (98% purity) was immediately dissolved in 0.1 M sodium phosphate buffer (pH 7.4) that contained a preservative of 0.02% sodium azide. The following glycation agents were used: glucose (Glc), fructose (Fru), and galactose (Gal). In addition, aldehydes, glyoxal (GO), and methylglyoxal (MGO) were utilized. BSA was incubated with propranolol (1 mM) as well as 0.5 M of Glc, Fru, and Gal for 6 days or GO and MGO (2.5 mM) for a period of 12 h.^20−22,24−27^ GO and MGO were utilized within a month after delivery, and working solutions were assembled briefly before assessment. In order to study the antioxidant properties of propranolol, BSA with propranolol was incubated with 20 mM of chloramine T (ChT) for 1 h.^28^ All the samples were subjected to strict incubation conditions, including incubation in darkness, sealed vials, and continuous shaking at a speed of 50 rpm and at a temperature of 37 °C.^19,20,26^ All of the incubation mixtures achieved a final concentration of 0.09 mM BSA.

In order to differentiate the results obtained for propranolol, aminoguanidine as a known protein oxidation inhibitor and captopril as an established antioxidant were utilized. Exact concentrations of glycation agents and specific, optimal incubation conditions were determined and validated in compliance with the outcome of the previous kinetic studies.^19−25^ Despite this, the concentrations of oxidants, sugars, and aldehydes were substantially higher than the physiological reference values. They are instrumental in modeling the physiological processes that occur in the human body over weeks or even months in a notably short time.^19,20,26^ These conditions are routinely applied to determine the antiglycation properties of new substances.^19,20,26−31^ All the additives (1 mM) had their concentration determined in compliance with the outcome of other *in vitro* studies, in proportion to the high concentrations of the glycating agents.^19−25^ The study was conducted in three series, each duplicated.

### Products of Protein Glycation

#### 2.4.1 Amadori Products (APs)

A colorimetric nitroblue tetrazolium (NBT) assay was conducted to determine the total levels of APs. The monoformazan extinction coefficient, determined at 12,640 M^–1^ cm^–1^, allowed us to calculate the absorbance at a wavelength of 525 nm.^32^

#### 2.4.2 β-Amyloid (βA)

The fluorescence emitted during the binding of amyloid fibrils/oligomers to thioflavin T was analyzed. First, 10 μL of thioflavin T and 90 μL of samples were mixed on a microplate. The fluorescence was measured at a wavelength of 385/485 nm.^33^

#### 2.4.3 Advanced Glycation End Products (AGEs)

The content of AGEs was measured spectrofluorimetrically at a wavelength of 440/370 nm in a 96-well microplate reader.^34,35^ Before the study, H_2_SO_4_ (0.1 M, 1:5, v/v) had been used for diluting the samples.^36^

#### 2.4.4 Products of Glycoxidation

Dityrosine (DT), *N*-formylkynurenine (NFK), and kynurenine (KN) were assayed spectrofluorimetrically at the excitation/emission wavelengths of 365/480, 325/434, and 330/415 nm, respectively. Before the study, all the samples had been diluted with H_2_SO_4_ (0.1 M, 1:5, v/v).^36^ All the results were standardized to the fluorescence of quinine sulfate solution (0.1 mg/mL) in 0.1 M H_2_SO_4_.^37^

### Products of Protein Oxidation

#### 2.4.5 Protein Carbonyls (PCs)

In order to determine the concentration of PCs, 2,4-dinitrophenylhydrazine (2,4-DNPH) and carbonyls reacted in proteins damaged by oxidation. The absorbance of the reaction product was determined colorimetrically at a wavelength of 355 nm. The absorbance coefficient for 2,4-DNPH was used as a standard (22 000 M^–1^ cm^–1^).^38^

#### 2.4.6 Advanced Oxidation Protein Products (AOPPs)

A spectrophotometric assay was conducted to evaluate the concentration of the AOPPs. First, PBS was used for diluting the assayed samples (200 μL) in a 1:5 (v/v) ratio. The mixture and 0–100 μmol/L standard and blank PBS solutions (200 μL) were placed on a 96-well microplate. Next, 1.16 M potassium iodide (10 μL) (KI) as well as acetic acid (20 μL) (chem formula) was put into the wells. At a wavelength of 340 nm, the absorbance was calculated instantaneously in the microplate reader and compared with the blank solution (PBS (200 μL), potassium iodide (10 μL), acetic acid (20 μL)). The linear absorbance was represented (range: 0–100 μmol/L) by the ChT solutions.^34^

### Molecular Docking

Molecular docking is a clear *in silico* method of predicting the best-preferred position of a ligand postbinding with a macromolecule (commonly a protein). We examined the possible interaction of BSA, glycosidases (α-amylase (αA), α-glucosidase (αG), and sucrase-isomaltase (SI)), and AGE pathway proteins (RAGE, signal transducer and activator of transcription (STAT), Janus kinase 2 (JAK2), cAMP response element-binding protein (CREB), activating transcription factor 4 (ATF4), protein kinase RNA-like endoplasmic reticulum kinase (PERK), p38 mitogen-activated protein kinase (P38 MAPK), extracellular signal-regulated kinase (ERK), c-Jun N-terminal kinase (JNK), phosphoinositide-3-kinase (PI3-K), protein kinase beta (PKB/Akt2), C/EBP homologous protein (CHOP), nuclear factor-kB (NF-kB), RAF, rapidly accelerated fibrosarcoma 1 (RAF1), RAS-related 2 protein (RAS), and mechanic target of rapamycin (MTOR) with the propranolol molecule. The Protein Data Bank (PDB) Web site (https://www.rcsb.org/) was accessed to download a 3D crystal structure of BSA (ID: 4F5S)^39^ in the.pdb format. The protein structure was determined by means of X-ray diffraction at a resolution value of 2.47 Å.^39^ The National Library of Medicine Web site (https://pubchem.ncbi.nlm. nih.gov/) provided the 3D structure of propranolol (ID: 6882).^40^ In the beginning, AutoDock MGLTools^41^ allowed to remove all the water molecules and replace them with polar hydrogen and Kollman’s partial charges to minimize energy input. Next, the prepared protein structure was saved as a.pdbqt file. AutoDock Vina^42^ (grid size of 40 × 40 × 40, with 0.375 Å spacing, located at coordinates 34.885, 23.976, and 98.792) simulated the possible molecular docking. The exhaustiveness parameter was set at a level of 8. Finally, PyMOL 2.5 allowed us to visualize the possible molecular docking.^23,43−46,47−49^

### Statistical Analysis

GraphPad Prism 9.000 (GraphPad Software, San Diego, California, USA) allowed the statistical analysis to be completed. The results were conveyed in terms of the percentage share of the respective control values (BSA with glycation agents). The one-way analysis of variance (ANOVA) followed by Tukey’s *post hoc* test for multiple comparisons allowed to determine the differences between the groups, and *p* < 0.05 was found to be statistically significant. Furthermore, a multiplicity-adjusted *p*-value was also determined.

## 5 Results

### 5.1 Systematic Review

The systematic review of the bibliography allowed for 279 publications to be recognized from the Medline (PubMed) database, including 223 that were omitted due to their title. Tihrty-three out of 56 abstracts were in line with the inclusion and exclusion criteria. Out of the eligible papers, 15 were found not to be connected to the topic of our research. Nevertheless, 18 papers were finally included (Figure 2). The final results of our systematic review are listed in Table 2.

**Table 2:** Multidirectional Characteristics of Propranolol in Both Clinical and Experimental Studies

**study design**	**end points**	**references**
**In vitro studies**		
an enantioselective analytical technique was used for assessing the interaction of propranolol with α1-acid-glycoprotein (AGP) at the microliquid–liquid interface. AGP was added to aqueous solutions of propranolol hydrochloride	both cyclic voltammetry and differential pulse voltammetry current responses decreased. Enantioselective binding by AGP of (*S*)-propranolol (2.7 × 10^5^ M^–1^) was proven over (*S*)-propranolol, and (*R*)-propranolol (1.3 × 10^5^ M^–1^)	(50)
carboxy-terminal polypeptide (residues 121–231; PrP121–231)—only autonomous folding unit of PrP with a defined 3D structure. Utilizing a PrP-immobilized biosensor chip, mouse embryonic fibroblasts were isolated from	a higher response value was expressed by diazepam, promethazine, and propranolol than by quinine hydrochloride, a well-established effective antiprion compound. Propranolol was identified as a new antiprion compound.	(51)
QC13.5-day-old embryos of angiotensin II type 1 receptor (AT1aR)-deficient mice. It was done to analyze the interaction between the angiotensin receptor and the β-adrenergic receptor and its impact on the production of amyloid β-protein (βA).	the well-known increase in βA production after treatment with Telm actually decreased following the addition of propranolol in a dose-dependent manner. Therefore, the interaction between AT1R and the β-adrenergic receptor (β-AR) may play a role in the pathway that stimulates βA production caused by Telm.	(52)
assessing the βA-induced reduction of soluble amyloid precursor protein α (sAPPα) in SH-SY5Y neuroblastoma cells.	50 μM of propranolol ameliorated the βA-induced reduction of sAPPα secretion; therefore, proposing diacylglycerol (DAG) may account for the βA-induced reduction of sAPPα.	(53)
[3H] myristic acid-prelabeled LA-N-2 cells were exposed to various concentrations of amyloid beta protein^25−35^ ranging from 20 to 250 μg/mL, and the activation of phospholipases A and D was assessed. Various substances such as propranolol, 7-chlorokyneurenic acid, metabotropic amino acid antagonist, and [Tyr^4^-d-Phe^12^] bombesin were assessed for βA protein stimulation of the phospholipase C activity.	propranolol, 7-chlorokyneurenic acid, metabotropic amino acid antagonist, and [Tyr^4^-d-Phe^12^] bombesin were determined to decrease the βA protein stimulation of the phospholipase C activity inside the [^3^H]inositol-prelabeled LA-N-2 cells. Therefore, the βA protein activation of phospholipase C may be receptor-mediated.	(54)
**In vivo studies**		
2-month-old APPswe/PS 1dE9 mice served the purpose of the analysis whether the B-adrenoreceptor antagonist, propranolol, would impact fear memory persistence.	the intra-CA1 infusion of propranolol impaired long-term fear memory only when administered immediately before conditioning in their wild-type counterparts.	(55)
pretreatment with propranolol during intracerebroventricular (ICV) administration of amylin (1–100 pmol) or intravenous (IV) administration of amylin (1–100 pmol), or an amylin agonist, salmon calcitonin, induces energy expenditure in anesthetized rats.	pretreatment with propranolol (5 mg kg^–1^ (iv)) blocks all the effects produced by ICV or IV administration of amylin.	(56)
CELP male mice (25–28 g) were employed and pretreated with adrenergic, cholinergic, serotonergic, dopaminergic, opiate, and GABA-A antagonists, including propranolol, to assess what blocks the memory consolidating action of MZ-4-71.	propranolol did not influence the effects of MZ-4-71.	(57)
cholesterol ester transfer protein (CETP) and lipoprotein lipase (LPL)-deficient (CELP) male mice treated with MZ-4-71 (10 μG/2 μL i.c.v.) and assessment of the immobility, climbing and swimming time to analyze what mediates the antidepressant-like events of MZ-4-71.	propranolol did not influence the effects of MZ-4-71.	(58)
the analysis of agonists (isoproterenol and salbutamol) and antagonists (propranolol and carvedilol) of beta-adrenoreceptors in ovariectomized rats to test for the effects on the hippocampal neurons by means of immunohistochemistry assays.	beta-adrenoreceptor antagonist propranolol regulated the effect of hormone diminishment, improved memory, and diminishment of neuronal death as well as βA-related changes in certain regions (e.g., cornu Ammonis regions (CA1–3) and dentate gyrus) of rat hippocampus.	(59)
analyzing whether l-3-*n*-butylphthalide (NBP) affects the glymphatic clearance and βA deposit in APP/PS_1_ mice.	propranolol inhibited the perivascular drainage of βA via increased cerebral pulsation. NBP affects the glymphatic clearance and βA deposit in APP/PS_1_ mice, proposing that it may have potential in the treatment of Alzheimer’s disease.	(60)
mice were pretreated with various substances, including propranolol, to assess the impact of neuropeptide AF (NPAF) on passive avoidance learning.	NPAF improves the consolidation of passive avoidance learning. Propranolol reversed the action of NPAF.	(61)
assessment of microglial protection by rodent’s environmental enrichment. Analyzing microglial morphology and inflammatory RNA profiles to prove if β-adrenergic signaling is the key.	mice in environmental enrichment after being fed with propranolol lost microglial protection against βA.	(62)
postmortem entorhinal cortex analysis of AD patients	propranolol decreased the fibril βA42-induced α-amylase activity. α-amylase is a vital factor associated with AD neuroinflammation	(63)
propranolol (5 mg/kg) tested in a model of chronic corticosterone administration (100 μg/mL, 4 weeks) in 32 male AKR/J mice.	propranolol diminished cognitive deficits, βA levels, tau phosphorylation, and insulin resistance in acknowledgment of chronic corticosterone administration.	(64)
16 male senescence-accelerated mouse prone 8 (SAMP8) and 16 senescence-accelerated mouse resistant 1 (SAMR1) mice were treated with propranolol, and assessment of memory deficits in SAMP8 was assessed	propranolol reduced cognitive memory impairments in SAMP8 mice in NORT. Propranolol reversed amyloid, Tau, and synaptic pathology in SAMP8 mice.	(65)
16 female Tg2576 AD transgenic mice were treated with propranolol once daily (5 mg/kg) or saline for 6 consecutive weeks. In the last week, a novel object recognition test (NORT) and fear conditioning test were performed. In addition, behavioral tests, object recognition tests, fear conditioning tests and neuronal primary culture tests were performed.	propranolol was shown to diminish cognitive deficits, amyloid and Tau pathology in Alzheimer’s transgenic mice.	(66)
**Ex vivo studies**		
on postnatal days 1–3, rat pups were used for culturing dissociated astrocytes from cortices. Confluent astrocytes were used for the purpose of pharmacological studies. Western and Northern blots were conducted for analysis purposes.	propranolol blocked the increase in amyloid precursor protein (APP) mRNA and holoprotein levels during the treatment of astrocytes with norepinephrine or isoproterenol for 24 h.	(67)

### 5.2 Scavenging of ROS and Total Oxidant Properties

ROS are products of enzymatic and nonenzymatic reactions of oxidative metabolism. Those chemically active molecules, despite low concentrations, participate in many physiological processes. Increased ROS concentrations lead to oxidative alterations of the cellular biomolecules. Antioxidant properties of the test sample may be determined by studying the scavenging capacity of hydrogen peroxide (H_2_O_2_), hydroxyl radical (HO•), and 2,2-diphenyl-1-picrylhydrazyl (DPPH•).^68,69^

#### 3.2.1 Scavenging of H_2_O_2_

**Figure 3:**
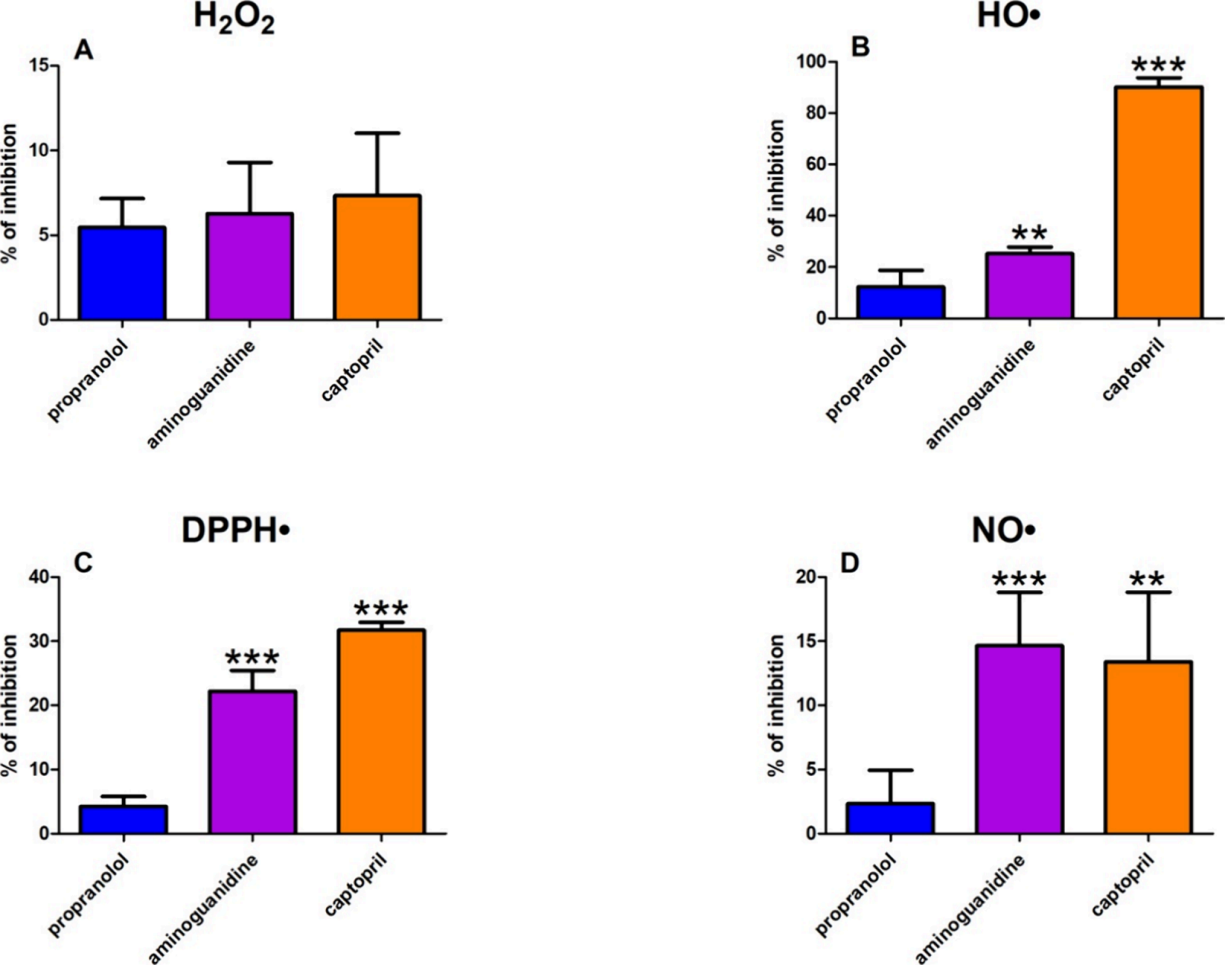
Impact of propranolol and other additives on the scavenging capacity of hydrogen peroxide (H_2_O_2_), hydroxyl radical (HO•), 2,2-diphenyl-1-picrylhydrazyl radical (DPPH•), and nitric oxide radical (NO•). AG: aminoguanidine; CPT: captopril; PPN: propranolol; H_2_O_2_: hydrogen peroxide; HO•: hydroxyl radical; NO• nitric oxide; DPPH•: 2,2-diphenyl-1-picrylhydrazyl radical. **p < 0.01 versus control (propranolol); ***p < 0.001 versus control (propranolol).

Propranolol scavenged H_2_O_2_ at a rate of 5% in the assay. There were no meaningful differences in the inhibition rate of H_2_O_2_ scavenging in comparison to that of propranolol (Figure 3A).

#### 3.2.2 Scavenging of HO•

HO**•** scavenging capacity of propranolol was 12%, whereas the inhibition rate of H_2_O_2_ scavenging of aminoguanidine (+1468%, *p* < 0.01) and captopril (+634%, *p* < 0.001) was much higher (Figure 3B).

#### 3.2.3 Scavenging of DPPH•

DPPH• scavenging capacity of AG and captopril was notably elevated (+417%, *p* < 0.001, and +638%, *p* < 0.001, respectively) as compared to propranolol (4%) (Figure 3C).

#### 3.2.4 Scavenging of NO• Radical

Propranolol scavenged NO• at a rate of 2% in the assay. The inhibition rate of NO• scavenging of AG (+526%, *p* < 0.001) and captopril (+472%, *p* < 0.01) was considerably higher than the inhibition rate of propranolol (2%) (Figure 3D).

### 5.3 FIC

**Figure 4:**
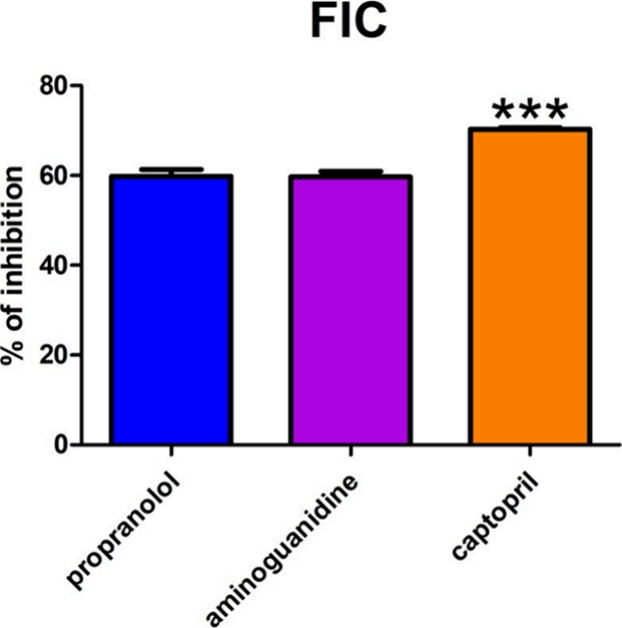
The impact of propranolol and other additives on ferrous iron chelating (FIC). AG: aminoguanidine; CPT: captopril; PPN: propranolol; FIC: ferrous iron chelating. ***p < 0.001 versus control (propranolol).

The FIC of propranolol was 60%. That parameter markedly decreased in captopril (17%, *p* < 0.001) (Figure 4).

### 5.4 Protein Glycation Products

The sequence of chemical reactions between proteins and reducing sugars through a nonenzymatic pathway is also known as the Maillard reaction. First of all, the glycation process may be initiated by reducing sugars covalently attached to the amino groups of proteins to produce a Schiff base. The unstable Schiff base may be transformed into more stable APs that will later undergo dehydration and rearrangement with AGE formation. Extended exposure of proteins to Glc and other sugars results in the α-helix transition into a linear structure, activating the formation of βA.^70−72^

### APs

**Figure 5:**
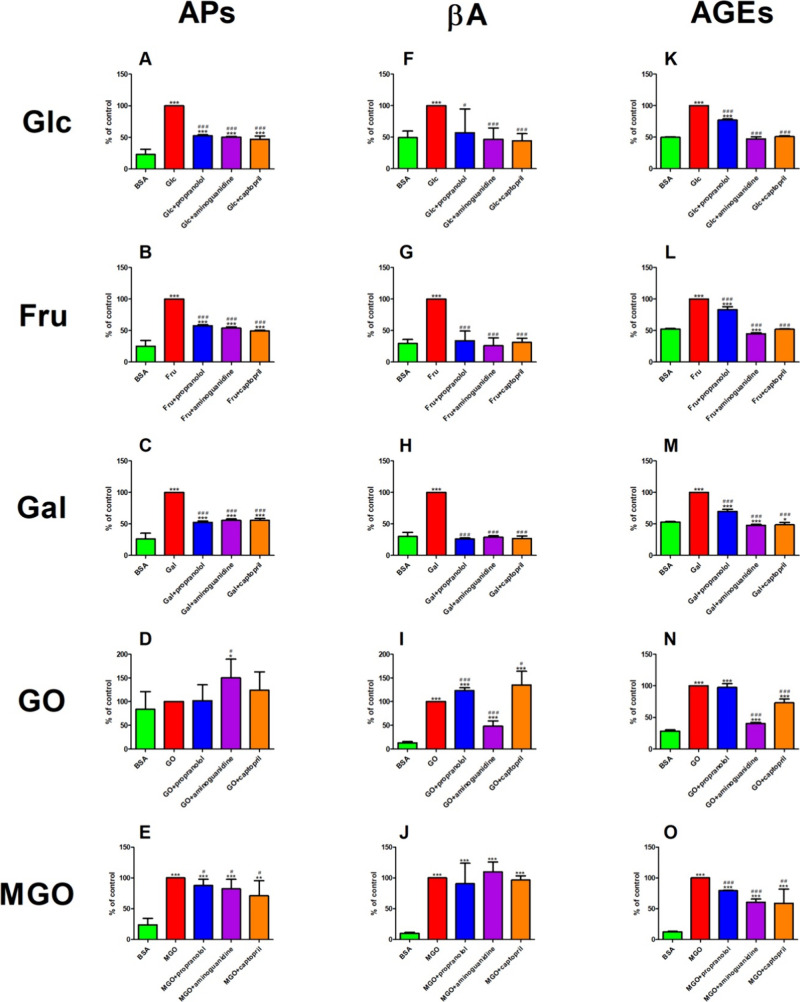
Impact of propranolol and other additives on protein glycation products in various glycation models. AGEs: advanced glycation end products; APs: Amadori products; BSA: bovine serum albumin; βA: b-amyloid; CPT: captopril; Fru: fructose-induced albumin glycation; Gal: galactose-induced albumin glycation; Glc: glucose-induced albumin glycation; GO: glyoxal-induced albumin glycation; MGO: methylglyoxal-induced albumin glycation; *p < 0.05 vs positive control (glycation/oxidizing agent); **p < 0.01 vs positive control (glycation/oxidizing agent); ***p < 0.001 vs positive control (glycation/oxidizing agent); ^#^*p* < 0.05 vs negative control (BSA); ^##^*p* < 0.01 vs negative control (BSA); ^###^*p* < 0.001 vs negative control (BSA).

The fluorescence of APs was suppressed in Glc+propranolol (−48%), Glc+aminoguanidine (−50%), and Glc+captopril (−53%) compared to Glc. The concentration of APs was effectively elevated in Glc+propranolol (+128%), Glc+aminoguanidine (+118%), and Glc+captopril (+104%) as compared to BSA. The biomarker was effectively enhanced in Glc (+334%) as compared to BSA (Figure 5A).

The fluorescence of APs decreased in Fru+propranolol, Fru+aminoguanidine, and Fru+captopril (−42, −46, and −51%, respectively) versus Fru. The level of APs was meaningfully attenuated in Fru, Fru+propranolol, Fru+aminoguanidine, and Fru+captopril (+298, +129, +114, and +96%, respectively) compared to Fru (Figure 5B).

The concentration of APs was markedly reduced in Gal+propranolol (−48%), Gal+aminoguanidine (−44%), and Gal+captopril (−44%) versus Fru. On the other hand, the level of APs was efficiently potentiated in Gal+propranolol, Gal+aminoguanidine, and Gal+captopril (+102, + 115, and +114%) as compared to BSA. The marker relatively increased in Gal (+286%) as compared to Gal (Figure 5C).

The level of APs was relevantly enhanced in GO+aminoguanidine (+50%) as compared to GO. On the other hand, the concentration of APs effectively increased in GO+aminoguanidine (+79%) as compared to BSA (Figure 5D).

The concentration of APs relevantly diminished in MGO+propranolol (−12%), MGO+aminoguanidine (−18%), and MGO+captopril (−29%) versus MGO. However, the level of APs was meaningfully potentiated in MGO+propranolol, MGO+aminoguanidine, and MGO+captopril (+267, + 245, and +197%, respectively) compared to BSA. The parameter was effectively elevated in MGO (+318%) compared to BSA (Figure 5E).

### β-Amyloid (βA)

The fluorescence of βA relevantly diminished in Glc+propranolol, Glc+aminoguanidine, and Glc+captopril (−43, −53, and −56%, respectively) compared to Glc. However, the bA content was markedly elevated in Glc (+104%) as compared to BSA (Figure 5F).

The content of βA diminished in Fru+propranolol (−66%), Fru+aminoguanidine (−74%), and Fru+captopril (−69%) versus Fru. On the other hand, the fluorescence of βA substantially increased in Fru (+238%) as compared to BSA (Figure 5G).

The fluorescence of βA markedly diminished in Gal+propranolol, Gal+aminoguanidine, and Gal+captopril (−74, −71, and −73%, respectively) versus Gal. However, the βA content was enhanced in Gal (+232%) versus BSA (Figure 5H).

The fluorescence of βA was augmented in GO+propranolol and GO+captopril (+23 and +35%, respectively) versus GO. That parameter was markedly reduced in GO+aminoguanidine (−52%) as compared to GO. On the other hand, the content of βA was significantly improved in GO (+670%), GO+propranolol (+847%), GO+aminoguanidine (+270%), and GO+captopril (+941%) as compared to BSA (Figure 5I).

The content of βA was relevantly enhanced in MGO (+911%), MGO+propranolol (+815%), and MGO+captopril (+875%) versus BSA. That parameter was higher in MGO+aminoguanidine (+1011%) versus BSA (Figure 5J).

### AGEs

The fluorescence was relevantly attenuated in Glc+propranolol (−23%) versus Glc. That parameter efficiently decreased in Glc+aminoguanidine and Glc+captopril (−53 and −49%, respectively) versus Glc. However, the content of AGEs was elevated in Glc+propranolol (+55%) versus BSA. That parameter was improved in Glc (+101%) versus BSA (Figure 5K).

The content of AGEs was meaningfully lowered in Fru+propranolol, Fru+aminoguanidine, and Fru+captopril (−17, −55, and −48%, respectively) versus Fru. On the other hand, the fluorescence of AGEs markedly diminished in Fru+aminoguanidine (−15%) as compared to BSA. That parameter was substantially enhanced in Fru (+91%) and Fru+propranolol (+59%) versus BSA (Figure 5L).

The content of AGEs meaningfully diminished in Gal+propranolol (−30%), Gal+aminoguanidine (−52%), and Gal+captopril (−51%) versus Gal. The fluorescence of AGEs relevantly decreased in Gal+aminoguanidine (−10%) and Gal+captopril (−8%) as compared to BSA. That parameter markedly increased in Gal (+89%) and Gal+propranolol (+32%) as compared to BSA (Figure 5M).

The content effectively diminished in GO+captopril (−27%) versus GO. That parameter was alleviated more effectively in GO+aminoguanidine (−60%) than in the case of GO. The fluorescence of AGEs was enhanced in GO+aminoguanidine (+43%) as compared to that in BSA. That parameter was significantly elevated in GO (+255%), GO+propranolol (+246%), and GO+captopril (+160%) as compared to that in BSA (Figure 5N).

The content of AGEs was significantly lower in MGO+propranolol (−21%), MGO+aminoguanidine (−39%), and MGO+captopril (−41%) versus MGO. However, the fluorescence ofAGEs was effectively elevated in MGO+aminoguanidine (+383%) and MGO+captopril (+368%) compared to BSA. That biomarker was relevantly augmented in MGO (+696%) and MGO+propranolol (+531%) versus BSA (Figure 5O).

### 5.8 Protein Glycoxidation Products

#### DT

**Figure 6:**
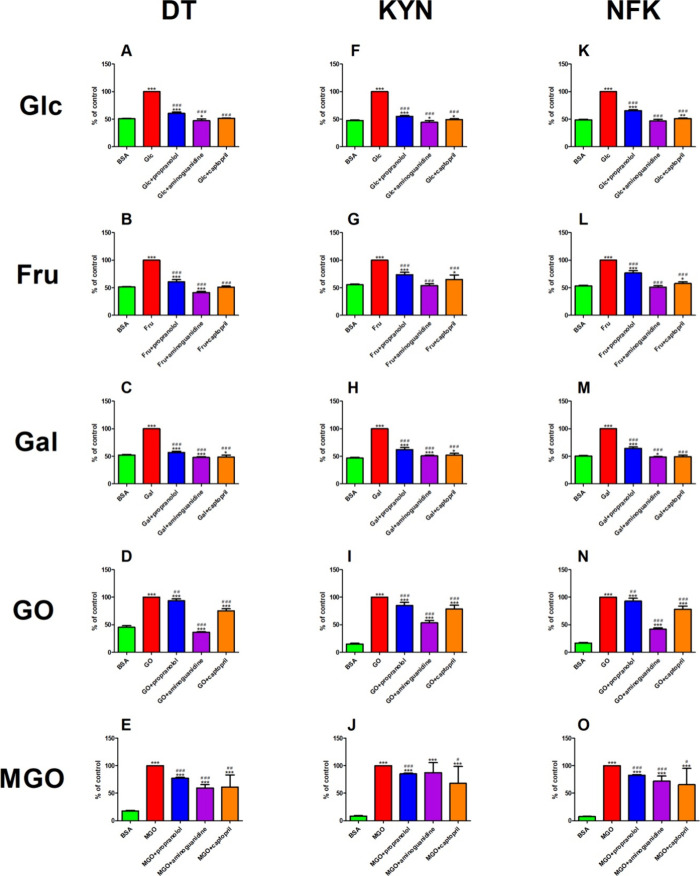
Effect of propranolol and other additives on protein glycoxidation products in various glycation models. BSA: bovine serum albumin; CPT: captopril; Fru: fructose-induced albumin glycation; Gal: galactose-induced albumin glycation; Glc: glucose-induced albumin glycation; GO: glyoxal-induced albumin glycation; MGO: methylglyoxal-induced albumin glycation; *p < 0.05 vs positive control (glycation/oxidizing agent); **p < 0.01 vs positive control (glycation/oxidizing agent); ***p < 0.001 vs positive control (glycation/oxidizing agent); ^#^*p* < 0.05 vs negative control (BSA); ^##^*p* < 0.01 vs negative control (BSA); ^###^*p* < 0.001 vs negative control (BSA).

The content of DT was effectively reduced in Glc+propranolol (−39%), Glc+aminoguanidine (−53%), and Glc+captopril (−48%) as compared with Glc alone. On the other hand, the DT fluorescence was meaningfully reduced in Glc+aminoguanidine (−7%) versus BSA. That biomarker was markedly elevated in Glc (+97%) and Glc+propranolol (+19%) in comparison with BSA (Figure 6A).

The production of DT was substantially mitigated in propranolol (−39%), Fru+aminoguanidine (−59%), and Fru+captopril (−49%) compared to that in Fru. However, the fluorescence of DT was effectively lowered in Fru+aminoguanidine (−19%) versus BSA. That parameter markedly increased in Fru (+95%) and Fru+propranolol (+18%) as compared to BSA (Figure 6B).

The DT fluorescence was effectively suppressed in propranolol (−43%), Gal+aminoguanidine (−52%), and Gal+captopril (−52%) as compared to Gal. The fluorescence of DT was relevantly reduced in Gal+aminoguanidine (−8%) and Gal+captopril (−8%) versus BSA. That parameter was meaningfully enhanced in Gal (+91%) and Gal+propranolol (+9%) in comparison with BSA (Figure 6C)

The DT fluorescence was relevantly lowered in propranolol (−7%), GO+aminoguanidine (−64%), and GO+captopril (−25%) as compared to GO. On the other hand, the content of DT was efficiently suppressed in GO+aminoguanidine (−20%) versus BSA. That parameter was enhanced in GO (+121%), GO+propranolol (+106%), and GO+captopril (+66%) when compared to BSA (Figure 6D).

The DT level was markedly attenuated in propranolol (−23%), MGO+aminoguanidine (−41%), and MGO+captopril (−39%) versus MGO. On the other hand, the fluorescence of DT was substantially augmented in MGO, MGO+propranolol, MGO+aminogunidine, and MGO+captopril (+467, +337, +236, and 246%, respectively) versus BSA (Figure 6E).

#### KYN

The content of KN was relevantly inhibited in propranolol (−45%), Glc+aminoguanidine (−56%), and Glc+captopril (−51%) compared to Glc. The fluorescence of KYN was markedly attenuated in Glc+aminoguanidine (−6%) as compared to BSA. That biomarker was effectively potentiated in Glc+propranolol (+16%) and Glc+captopril (+4%) versus BSA. The KN fluorescence was boosted in Glc (+111%) versus BSA (Figure 6F).

The KYN content markedly decreased in Fru+propranolol (−26%), Fru+aminoguanidine (−46%), and Fru+captopril (−35%) versus Fru. However, the fluorescence of KYN was augmented in Fru (+80%), Fru+propranolol (+33%), and Fru+captopril (+17%) in comparison with BSA (Figure 6G).

The KYN fluorescence significantly diminished in propranolol (−38%), Gal+aminoguanidine (−49%), and Gal+captopril (−48%) in comparison with that in Fru alone. The content of KYN was elevated in Gal+aminoguanidine (+8%) and Gal+captopril (+10%) versus BSA. That biomarker was meaningfully elevated in Gal (+113%) and Gal+propranolol (+32%) in comparison with BSA alone (Figure 6H).

The fluorescence of GO was significantly lower in propranolol (−15%), GO+aminoguanidine (−47%), and GO+captopril (−22%) versus GO. On the other hand, the content of GO was significantly elevated in GO (+579%), GO+propranolol (+476%), GO+aminoguanidine (+263%), and GO+captopril (+433%) versus BSA (Figure 6I).

The production of MGO was suppressed in propranolol (−15%) and MGO+captopril (−32%) as compared to MGO. The content of MGO was significantly enhanced in MGO (+1081%), MGO+propranolol (+905%), MGO+aminoguanidine (+930%), and MGO+captopril (+701%) versus BSA (Figure 6J).

#### NFK

The production of NFK substantially decreased in propranolol (−45%), Glc+aminoguanidine (−56%), and Glc+captopril (−49%) in comparison to Glc. The fluorescence of NFK was enhanced in Glc+captopril (+4%) versus BSA. That biomarker was relevantly attenuated in Glc (+105%) and Glc+propranolol (+34%) in comparison with BSA (Figure 6K).

The NFK fluorescence was effectively reduced in propranolol (−23%), Fru+aminoguanidine (−49%), and Fru+captopril (−42%) versus Fru. However, the content of NFK was elevated in Fru+captopril (+8%) as compared to BSA. That biomarker was relevantly enhanced in fructose (+87%) and Fructose+propranolol (+44%) versus BSA (Figure 6L).

The NFK fluorescence relevantly diminished in propranolol (−36%), Gal+aminoguanidine (−51%), and Gal+captopril (−51%) in comparison to that in Gal alone. On the other hand, the content of NFK was meaningfully reduced in Gal+aminoguanidine (−3%) versus BSA. That parameter was efficiently enhanced in galactose (+98%) and Gal+propranolol (+28%) versus BSA (Figure 6M).

The fluorescence of NFK was lowered in propranolol (−7%) versus GO. That parameter was meaningfully diminished in GO+aminoguanidine (−58%) and GO+captopril (−22%) as compared to GO. The content of NFK was relevantly potentiated in GO (+510%), GO+propranolol (+466%), GO+aminoguanidine (+156%), and GO+captopril (+377%) in comparison with BSA (Figure 6N).

The NFK fluorescence relevantly diminished in propranolol (−18%), MGO+aminoguanidine (−28%), and MGO+captopril (−35%) versus MGO. However, the content of NFK markedly increased in MGO+propranolol (+999%), MGO+aminoguanidine (+856%), and MGO+captopril (+772%) versus BSA. That parameter was substantially boosted in MGO (+1234%) as compared to BSA (Figure 6O).

### 5.9 Protein Oxidation Products

Amino acids with free amino, amide, or hydroxyl groups (e.g., arginine (ARG), lysine (LYS), and TRY) may oxidize to produce PCs. AOPPs are the final products of the multifaceted process of protein oxidation. AOPPs are aggregates, fragments, or oxidatively altered albumin, fibrinogen, or lipoproteins derivatives. AOPPs molecules may hold DT, PCs, and modified excess of TRY, TYR, ARG, LYS, and amino acids, including sulfur.^73,74^

### PCs

**Figure 7:**
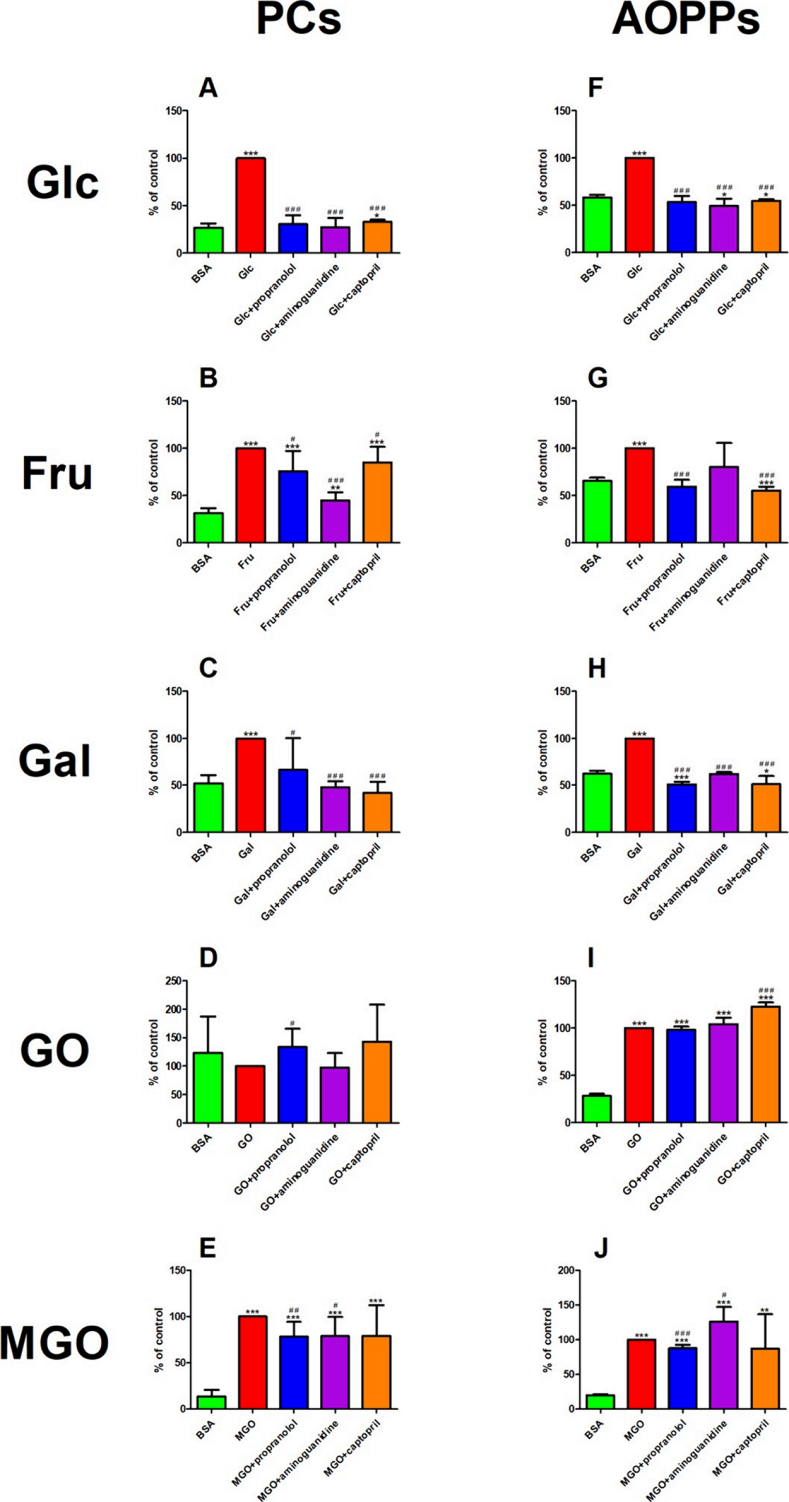
The effect of propranolol and other additives on protein oxidation products in various glycation models. AOPPs: advanced oxidation protein products; BSA: bovine serum albumin; CPT: captopril; Fru: fructose-induced albumin glycation; Gal: galactose-induced albumin glycation; Glc: glucose-induced albumin glycation; GO: glyoxal-induced albumin glycation; MGO: methylglyoxal-induced albumin glycation; PCs: protein carbonyls *p < 0.05 vs positive control (glycation/oxidizing agent); **p < 0.01 vs positive control (glycation/oxidizing agent); ***p < 0.001 vs positive control (glycation/oxidizing agent); ^#^*p* < 0.05 vs negative control (BSA); ^##^*p* < 0.01 vs negative control (BSA); ^###^*p* < 0.001 vs negative control (BSA).

The concentration of PCs markedly decreased in propranolol (−69%), Glc+aminoguanidine (−73%), and Glc+captopril (−67%) in comparison with Glc. However, the PC level increased in Glc+captopril (+22%) versus BSA. That biomarker was relevantly boosted in Glc (+274%) as compared to BSA (Figure 7A).

The PC concentration relevantly diminished in propranolol (−69%), Fru+aminoguanidine (−55%), and Fru+captopril (−15%) versus Fru. On the other hand, the concentration of PCs was enhanced in Fru+aminoguanidine (+42%) as compared to BSA. The biomarker was relevantly enhanced in Fru (+217%), Fru+propranolol (+140%), and Fru+captopril (+169%) versus BSA (Figure 7B).

The level of PCs diminished in propranolol (−34%), Gal+aminoguanidine (−52%), and Gal+captopril (−58%) in comparison with Gal. The PC concentration was effectively improved in Gal (+94%) versus BSA (Figure 7C).

The PC level was improved in propranolol (+33%) as compared to that in GO (Figure 7D).

The PC concentration substantially decreased in propranolol (−21%) and MGO+aminoguanidine (−21%) as compared to MGO. However, the level of PCs was markedly improved in MGO (+632%), propranolol (+475%), MGO+aminoguanidine (+480%), and MGO+captopril (+478%) versus BSA (Figure 7E).

### AOPPs

The concentration of AOPPs was markedly lowered in propranolol (−47%), Glc+aminoguanidine (−73%), and Glc+captopril (−67%) versus Glc. The level of AOPPs was meaningfully reduced in Glc+aminoguanidine (−16%) and Glc+captopril (−6%) compared to that in BSA. The biomarker was relevantly improved in Glc (+72%) versus BSA (Figure 7F).

The AOPP concentration significantly diminished in propranolol (−41%) and Fru+captopril (−45%) compared to Fru. The content of AOPPs was meaningfully reduced in Fru+captopril (−16%) versus BSA. The parameter was potentiated in Fru (+53%) as compared to BSA (Figure 7G).

The level of AOPPs effectively decreased in Gal+propranolol (−36%), Gal+aminoguanidine (−38%), and Gal+captopril (−49%) versus Gal. The AOPP concentration was efficiently inhibited in Gal+propranolol (−18%) and Gal+captopril (−18%) as compared to BSA. The biomarker was relevantly improved in Gal (+61%) versus BSA (Figure 7H).

The concentration of AOPPs was relevantly enhanced in GO+captopril (+23%) as compared with GO. The level of AOPPs was meaningfully potentiated in GO (+257%), GO+propranolol (+249%), GO+aminoguanidine (+271%), and GO+captopril (+337%) versus BSA (Figure 7I).

The level of AOPPs was significantly lower in MGO+propranolol (−12%) as compared to MGO. The biomarker was effectively improved in MGO+aminoguanidine (+26%) versus BSA. The AOPP concentration was substantially enhanced in MGO (+408%), MGO+propranolol (+385%), MGO+aminoguanidine (+540%), and MGO+captopril (+342%) in comparison with BSA (Figure 7J).

### Binding Affinity Analysis

The ability of propranolol to impede protein glycoxidation was also assessed in *in silico* studies. The molecular docking simulation between BSA and propranolol revealed its binding solid affinity, 7.8 kcal/mol. Just two docking sites had root-mean-square deviations of atomic positions (RMSD) below 3 (Table 3); however, only mode 2 revealed a polar contact with TYR-160 of the BSA particle. Mode 2 is presented in Table 3.

**Table 3:** Results of Molecular Docking Simulations between Propranolol and BSA^a^

**mode**	**affinity**(kcal/mol)	**RMSD (lower bond)**	**RMSD (upper bond)**	**amino acid residues**
1	–8.0	0.000	0.000	
2	–7.9	1.810	2.892	TYR-160
3	–7.5	25.500	28.502	
4	–7.2	24.666	27.204	
5	–7.2	24.189	27.194	
6	–7.2	23.942	26.997	
7	–7.1	22.134	24.583	LYS-136, GLU-140
8	–7.1	25.318	27.459	
9	–7.1	4.750	7.252	GLU-125

a Glu: glucose; LEU: leucine; LYS: lysine; RMSD: root-mean-square deviations of atomic positions; TYR: tyrosine.

**Figure 8:**
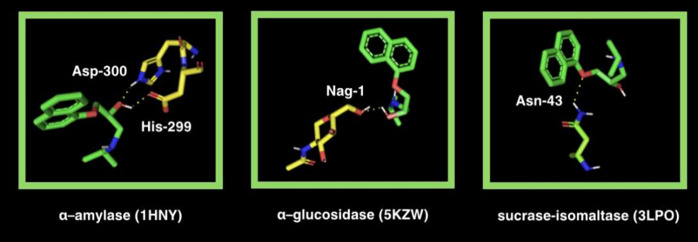
Visualization of propranolol docking sites in glycosidases: α-amylase (αA), α-glucosidase (αG), and sucrase-isomaltase (SI). Propranolol’s spatial structure has been marked in green color. ASN, asparagine; ASP, aspartic acid; NAG-1, *N*-acetylglucosamine.

The molecular docking was also performed between propranolol and glycosidases (α-amylase (αA), α-glucosidase (αG), and sucrase-isomaltase (SI)) and revealed strong binding affinities (−6.8, −6.7, and −6.0 kcal/mol). We have demonstrated that the most energetically favorable binding site is stabilized by two polar interactions (ASP-300 and HIS-299) (Table 4 and Figure 8).

**Table 4:** Results of Molecular Docking Simulations Conducted between Propranolol and Glycosidases^a^

**enzyme number**	**name of enzymes (EC number)**	**RCSB ID**	**affinity**(kcal/mol)	**number of polar contacts**	**amino acid residues**
1	α-amylase (αA; EC 3.2.1.1) 1HNY-7.0 1	1HNY	–6.8	2	ASP-300, HIS-299
2	α-glucosidase (αG; EC 3.2.1.20) 5KZW-6.3 4	5KZW	–6.7	1	NAG-1
3	sucrase-isomaltase (IS; EC 3.2.1.10) 3LPO-6.0 2	3LPO	–6.0	1	ASN-43

a ASN, asparagine; ASP, aspartic acid; HIS, histidine; NAG, *N*-acetylglucosamine.

**Figure 9:**
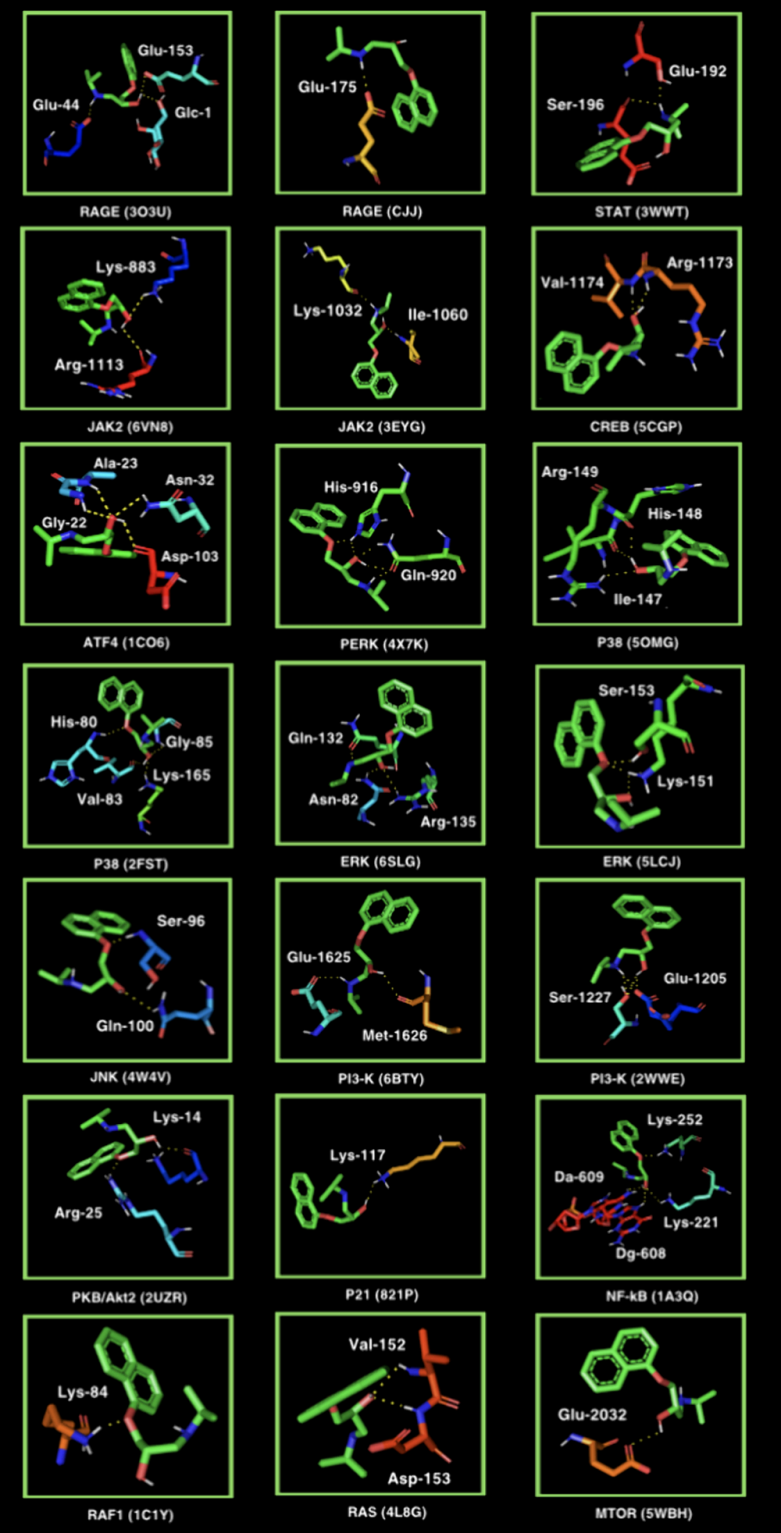
Visualization of propranolol docking sites in advanced glycation end products (AGEs) pathway proteins. Propranolol’s spatial structure has been marked in green color ALA, alanine; ARG, arginine; ASN, asparagine; ASP, aspartic acid; ATF4, activating transcription factor 4; CREB, cAMP response element-binding protein; DA, diaminopimelic acid; DG, d-glyceraldehyde; ERK, extracellular signal-regulated kinase; GLC, glycine; GLN, glutamine; GLU glutamic acid; GLY, glycine; HIS, histidine; ILE, isoleucine; JAK2, Janus kinase 2; JNK, c-Jun N-terminal kinase, LYS, lysine; MET, methionine; nF-kB, nuclear factor-kB; PERK, protein kinase RNA-like endoplasmic reticulum kinase; RAGE, receptor for advanced glycation end products; RAF, rapidly accelerated fibrosarcoma 1; RAS, RAS-related 2 protein; SER, serine; STAT, signal transducer and activator of transcription; VAL, valine.

The molecular docking for AGE pathway proteins indicated propranolol to have antiglycative properties *in vivo*, as it demonstrated the drug’s ability to bind to all the tested ligands, from −5.2 kcal/mol for cyclin-dependent kinase inhibitor 1 (P21). Outstandingly high binding affinity was highlighted for NF-kB, PI3-K, and MTOR (−7.4, −7.2, and −7.2 kcal/mol, respectively) (Table 5 and Figure 9). Molecular docking analysis did not exhibit any polar contact for CREB (4NYX), P38 MAPK (2FSL), or CHOP (3T92). Propranolol’s ability to bind to the AGE pathway may prevent adverse complications such as insulin resistance, inflammation, or the production of ROS, which aggravate protein glycation (Table 5 and Figure 9).

**Table 5:** Results of Molecular Docking Simulations between Propranolol and Advanced Glycation End Product (AGE) Pathway Proteins^a^

**name of protein**	**RCSB ID**	**affinity**(kcal/mol)	**number of polar contacts**	**amino acid residues**
*Receptors and signal transduction*				
RAGE	2GLX3O3U	–5.9	3	GLU-44, GLU-153, GLC-1
	2GLX3CJJ	–5.8	1	GLU-175
STAT	3WWT	–6.2	2	GLU-192, SER-196
JAK2	6VN8	–6.9	2	ARG-1113, LYS-883
	3EYG	–5.5	2	ILE-1060, LYS-1032
*Cellular stress response and apoptosis*				
CREB	4NYX	–6.0	0	
	5CGP	–5.2	2	ARG-1173, VAL-1174
ATF4	1CO6	–5.5	4	ALA-23, ASN-32, ASP-103, GLY-22
PERK	4 × 7K	–6.7	2	GLN-920, HIS-916
*Mitogen-activated protein kinases (MAPKs)*				
P38 MAPK	5OMG	–6.3	3	ARG-149, HIS-148, ILE-147
	2FST	–5.9	4	GLY-85, HIS-80, LYS-165, VAL-83
	2FSL	–5.8	0	
ERK	6SLG	–6.3	3	ARG-135, ASN-82, GLN-132
	5LCJ	–6.3	2	LYS-151, SER-153
JNK	4H39	–6.0	0	
	4W4 V	–6.0	2	GLN-100, SER-96
*Cell-cycle and antiapoptotic proteins*				
PI3–K	6BTY	–7.2	2	GLU-1625, MET-1626
	2WWE	–6.7	2	SER-1227, GLU-1205
PKB/Akt2	2UZR	–5.6	2	LYS-14, ARG-25
P21	821P	–5.2	1	LYS-117
*Stress response and apoptosis regulators*				
CHOP	3T92	–6.4	0	
NF-kB	1A3Q	–7.4	4	DA-609, DG-608, LYS-221, LYS-252
*Cell growth and proliferation*				
RAF1	1C1Y	–6.9	1	LYS-84
RAS	4L8G	–5.4	2	ASP-153, VAL-152
*Cellular signaling and protein synthesis*				
MTOR	5WBH	–7.2	1	GLU-2032

a ALA, alanine; ARG, arginine; ASN, asparagine; ASP, aspartic acid; ATF4, activating transcription factor 4; CHOP, C/EBP homologous protein; CREB, cAMP response element-binding protein; DA, diaminopimelic acid; DG, d-glyceraldehyde; ERK, extracellular signal-regulated kinase; GLC, glycine; GLN, glutamine; GLU glutamic acid; GLY, glycine; HIS, histidine; ILE, isoleucine; JAK2, Janus kinase 2; JNK, c-Jun N-terminal kinase, LYS, lysine; MAP, mitogen-activated protein kinase; MET, methionine; nF-kB, nuclear factor-kB; PERK, protein kinase RNA-like endoplasmic reticulum kinase; RAGE, receptor for advanced glycation end products; RAF, rapidly accelerated fibrosarcoma 1; RAS, RAS-related 2 protein; SER, serine; STAT, signal transducer and activator of transcription; P21, cyclin-dependent kinase inhibitor 1; P38 MAPK, p38 mitogen-activated protein kinase; PI3K, phosphoinositide-3-kinase; PKB/Akt2, protein kinase beta; VAL, valine.

## Discussion

Propranolol is a nonselective beta-adrenergic blocker that competitively blocks the response to beta_1_- and beta_2_-adrenergic stimulation. It causes a reduction in heart rate, blood pressure, myocardial contractility, and myocardial oxygen demand, making propranolol widely used in hypertension, tremor, angina, arrhythmia, and other cardiac or circulatory disorders.^9^ Although the drug has been used for decades, the pharmacological effects of propranolol are not fully understood—the related literature points to its potential antiglycoxidant activity.^75−78^

Our study is the first to comprehensively evaluate the antiglycation properties of propranolol using a glycated albumin model. Albumin, a major plasma protein, plays a vital role as a transport and buffering molecule.^79^ Nevertheless, albumin also has other biological properties. It can bind transition metal ions and endogenous and exogenous ligands such as hormones, inflammatory mediators, drugs, and pollutants. It also has a vigorous antioxidant activity.^80^ Not surprisingly, BSA is often used as a model protein because of its structural and functional similarities to human serum albumin.^81^ Within the framework of our study, several glycation factors (Glc, Fru, Gal, GO, and MGO) have been applied to induce BSA glycation. Various glycation factors have been found to be necessary to mimic physiological conditions in the human body. Glucose is a commonly used sugar due to its physiological importance; however, we have decided to use several sugars and aldehydes due to their different kinetics in glycation reactions.^82−86^ The wide variety of glycation products formed under those conditions mimics (to some extent) the complexity of glycation processes observed *in vivo.*

The first step of protein glycation is to form a Schiff base, the preliminary reaction between the carbonyl group of a reducing sugar/aldehyde and the amino group of a protein to form a reversible Schiff base.^87^ The Schiff base undergoes an irreversible rearrangement to produce an early glycation product known as the APs.^88^ Oxidation, dehydration, and rearrangement reactions transform APs into AGEs that are very stable protein structures.^72^ Both dicarbonyl compounds, methylglyoxal (MGO) and glyoxal (GO), are highly toxic due to their glycating solid abilities.^89^ MGO acts as a potent glycation agent by rapidly reacting with lysine and arginine residues in proteins to produce AGEs.^90^ Similarly, GO facilitates the glycation process by reacting with amino groups and initiating the formation of AGEs.^85^ It has been proven that the concentration of MGO and GO increases inside mitochondria under hyperglycemic conductions, which profoundly affects mitochondrial respiration.^91,92^

The contents of protein glycation (↑APs, ↑βA, ↑AGEs), glycoxidation (↑DT, ↑KYN, ↑NFK), and oxidation products (↑PCs, ↑AOPPs) were immensely increased in the BSA samples with the addition of all the glycation agents, in comparison to BSA without additives (Figures 3–7). The specific incubation conditions and reagent concentrations were chosen based on kinetic studies, further demonstrating the feasibility of sugars and aldehydes in an *in vitro* BSA model.

In all of the glycation models, the contents of protein glycation (↓APs, ↓βA, ↓AGEs), glycoxidation (↓DT, ↓KYN, and ↓NFK), and oxidation (↓PCs, ↓AOPPs) products were significantly lower under the influence of propranolol (Figures 3–7). Propranolol often restored them to the initial BSA levels and, sometimes (βA in Gal), even more effectively than the baseline. Only APs, βA, and AGEs in GO were not significantly altered by the drug. The antiglycation effect of propranolol is also supported by the results of a systematic literature review (Table 2). Although few clinical studies have been conducted so far, *in vitro* and animal studies show that the drug’s antiglycation properties are mainly due to the reduced production/modification of Aβ.

Protein glycoxidation inhibition is the key to the prevention of cardiovascular complications. nonenzymatic glycation plays a vital role in the development of diabetes and micro- and macrovascular disorders.^93,94^ In atherosclerosis, the accumulation of AGEs in blood vessels contributes to endothelial dysfunction and promotes the adhesion of inflammatory cells and lipids to the vessel walls.^95^ AGEs may also modify lipoproteins, which increases their susceptibility to oxidation.^96^ Long-term hyperglycemia significantly increases the rate of the AGE formation and accumulation.^97^ That impairs the mechanism of the AGE removal from the body by reducing the activity of glyoxalase and other proteolytic enzymes.^98^

Inhibition of BSA glycation by propranolol is also confirmed by *in silico* analyses. Foremost, the molecular docking between propranolol and BSA was conducted. The purpose was to assess propranolol’s affinity in respect of albumin’s binding sites and its ability to compete with or displace another substance. The simulation of propranolol manifested its very weak affinity with the BSA particle with a score of −8 kcal/mol. Next, the molecular docking was conducted between propranolol and selected glycosidases. α-Amylase (αA), α-glucosidase (αG), and sucrase-isomaltase (SI) play a crucial role as digestive enzymes and break apart polysaccharides.^99−101^ Propranolol proved to have low binding energy for all hydrolases above (−6.8, −6.3, and −6.0 kcal/mol) (Table 4 and Figure 8). It is a widely understood concept that as the energy of the ligand–receptor decreases, the docking improves and affinity increases.^102^ The positive affinity of propranolol with the enzymes indicates the potential for inhibiting their activity. Therefore, propranolol’s ability to decrease the sugars may be connected to its antiglycative properties. The αA particle’s docking site consisted of two interactions with propranolol through aspartate (ASP)-300 and histidine (HIS)-299. The α*G*′s particle’s docking site had one polar interaction with propranolol through *N*-acetylglucosamine (NAG)-1, and the sucrase-isomaltase (SI) docking site had one interaction with propranolol through asparagine (ASN)-43.

Finally, *in silico* docking was conducted for all of the AGE pathway proteins (Table 5 and Figure 9). The role of the AGEs/RAGE signaling in the modulation of gene transcription is closely associated with the development of type 2 diabetes and its related complications.^103^ Propranolol portrayed perfect binding affinity (not lower than −5.2 kcal/mol) for all 21 proteins. The drug bound most prominently to NF-kB (−7.4 kcal/mol; four polar contacts) as well as PI3-K (−7.2 kcal/mol; two polar contacts) and MTOR (−7.2 kcal/mol; one polar contact). The NF-kB overactivation may increase protein glycation, stimulate vascular cell adhesion molecule-1 (VCAM-1), and activate inflammatory cell adhesion to the vascular endothelium. Propranolol proved to have four polar contacts with NF-kB through deoxyadenosine (DA)-608, (DA)-609, LYS-22, and LYS-25. Propranolol’s interactions with various cellular regulators such as transcription factors, MAPKs, and cell cycle proteins may contribute to the preservation of the pancreatic β-cell function and insulin sensitivity and regulation of cellular responses to oxidative stress and abnormal protein synthesis induced by AGEs. In patients with diabetes, there is an increased expression of the AGE–RAGE signaling, directly contributing to the development of metabolic complications associated with diabetes.^94^

Well-established protein glycation inhibitors and antioxidants were utilized to compare propranolol’s potential to protect against carbonyl stress. The antiglycation effect of propranolol was comparable to the protein glycation (aminoguanidine) and oxidation (captopril) inhibitors, which successfully prevented the changes induced by glycating/oxidizing agents. Aminoguanidine prevents glycation due to a guanidinium group competing or scavenging dicarbonyls.^104^ It also exhibits a direct antioxidant activity. Captopril shows antioxidant properties by scavenging free radicals and increasing the activity of antioxidant enzymes, such as superoxide dismutase (SOD) and catalase. Captopril may also inhibit the formation of AGEs by interfering with MGO and GO, which are essential precursors to the AGE formation.

Propranolol is a propanolamine in which propran-2-ol is switched by a propran-2-ylamino group at position 1 and a napthalen-1-yloxy group at position 3.^105^ Therefore, propranolol contains an aryloxypropanolamine structure. In combination with propranolamine, the aryloxy ring promotes its binding to beta-adrenergic receptors, resulting in a competitive blockade of epinephrine and norepinephrine.^106^ In contrast, propranolol’s hydroxyl and secondary amino groups promote water solubility and metabolite formation during conjugation reactions in the liver.^107^ The OH group also promotes interactions with biological targets, such as beta-adrenergic receptors.^108^ That chemical structure may also account for propranolol’s antioxidant activity, thus explaining the drug’s antiglycation effect. The potential antioxidant properties of propranolol confirm the results of our study. Although propranolol poorly scavenges the DPPH or HO radical, the ability to neutralize hydrogen peroxide or bind iron ions in the FIC assay is comparable to captopril and aminoguanidine. Gomes et al. demonstrated that certain β-blockers, including propranolol, acted as effective ROS (O_2_^–^, H_2_O_2_, HO•, HOCl, and ROO•) and/or RNS (•NO and ONOO^–^) scavengers. They proposed that those effects could prevent cardiovascular complications, including hypertension and other comorbidities. The HO• scavenging activity for propranolol was significantly lower than that for labetalol and pindolol but higher than that for sotalol, timolol, atenolol, and metoprolol. Similarly, the results of the •NO scavenging assay proved that atenolol and pindolol had a higher scavenging capacity than propranolol and carvedilol, both of which did not reach 50% effect at the maximum tested concentrations (5000 and 50 μm, respectively). All the assayed compounds, except for timolol and labetalol, were able to scavenge peroxynitrite (ONOO^–^); however, propranolol and atenolol showed IC_50_ (half-maximal inhibitory concentration) of 1112 ± 232 and 2415 ± 278 μM (mean ± standard error of the mean (SEM)), respectively. In a concentration-dependent fashion, propranolol was able to delay the loss of fluorescence due to the ROO• dependent fluorescein oxidation.^109^

The available related literature also confirms the antioxidant effects of propranolol *in vivo*. The d-isomer of propranolol has been shown to reduce cardiac Fe uptake and inflammation and protect against oxidative stress and progressive cardiac dysfunction in rats overloaded with iron.^110,111^ Propranolol may also act as a “chain-breaking” antioxidant to protect cardiac membrane lipids from peroxidative damage, in addition to simple (reversible) xanthine oxidase (XOD) inhibition.^112^ On the other hand, chronic propranolol treatment strengthens the antioxidant barrier and protects against ischemia-reperfusion injury in isolated hearts of animals without β-blockade.^76^ Therefore, propranolol’s antioxidant properties may be due to its beta-blocking effects. By reduction of the activity of the sympathetic nervous system, propranolol mitigates oxidative stress by inhibiting catecholamines that generate free radicals. Ranasinghe et al. assessed the impact of propranolol on nitrosative stress and antioxidant potential in patients suffering from resistant hypertension. Serum nitrate and nitrite levels were significantly lower after 90 days of propranolol treatment. The serum total antioxidant capacity (AOC) also increased in the study group as compared to the placebo group.^75^

Our study has shown that propranolol has antiglycation properties in *in vitro* and *in silico* models. The additional effect of propranolol is comparable to that of known inhibitors of protein glycoxidation. Although further studies are required, the drug may be particularly indicated for people with cardiovascular disease and diabetes. Propranolol is a well-known drug with an established safety profile. The drug is toxic at plasma concentrations of more than 2 μg/mL, and mortality occurs at doses of more than 3 μg/mL.^113,114^ It should be remembered that propranolol is a very lipophilic beta blocker. It may easily cross the lipid cell membrane/blood–brain barrier and cause seizures in overdose cases. In diabetic patients, propranolol may also mask some of the symptoms of hypoglycemia, e.g., tachycardia and tremors.^113,114^

It should be mentioned that our study has a possible limitations. The BSA glycoxidation model simplifies the complex molecular interactions between proteins *in vivo*, which creates difficulties in transferring the results to more complex physiological models. Additional studies of animal and human models are needed for further analysis. Thus, our work provides a starting point for further research.

## Data Availability

The supporting data of this study is available from the corresponding author upon a reasonable request.
